# Lung microbiota, gut microbiota, and the lung-gut axis in lung cancer immunotherapy: a tripartite regulatory perspective

**DOI:** 10.3389/fimmu.2026.1907688

**Published:** 2026-10-02

**Authors:** Qin Chen, Jing Wang, Daqiang Sun, Jianwen Qin

**Affiliations:** 1 Department of Respiratory and Critical Medicine, Tianjin Chest Hospital, Tianjin, China; 2 Tianjin Key Laboratory of Cancer Prevention and Therapy, Department of Thoracic Oncology, Tianjin Cancer Institute & Hospital, Tianjin Medical University, Tianjin, China; 3 Department of Thoracic Surgery, Tianjin Chest Hospital, Tianjin, China

**Keywords:** gut microbiota, immunotherapy, intratumoral microbiota, lung cancer, lung microbiota, lung-gut axis

## Abstract

The gut microbiota is well established as a key modulator of systemic immunity, with growing evidence supporting its impact on lung cancer immunotherapy outcomes. However, the contributions of the lung microbiota and the recently recognized intratumoral microbiota remain less defined. This review synthesizes current evidence on the gut-lung axis, consolidating the tripartite regulatory network of gut, lung, and intratumoral microbiota in shaping the lung cancer immune microenvironment and immunotherapy response. We discuss how gut-derived metabolites such as indole-3-propionic acid may remotely modulate CD8^+^ T cell stemness, how lung microbiota locally influence alveolar macrophage polarization through pattern recognition receptor signaling, and how intratumoral microbiota have been implicated in STING pathway activation, Treg/M2 polarization, and PD-L1 expression. Although these microbial communities are conceptually linked through the lung-gut axis, the empirical evidence supporting their integrated network remains preliminary, and the clinical relevance of these interactions requires further investigation. Future research should focus on establishing causal relationships between specific microbial taxa and immunotherapy outcomes, elucidating spatial heterogeneity through multi-omics approaches, and validating microbiota-targeted interventions in well-designed prospective studies. This review offers a framework for critically evaluating the current evidence and identifying key knowledge gaps in this evolving field.

## 1 Introduction

Lung cancer remains the leading cause of cancer-related mortality worldwide (1). Immune checkpoint inhibitors (ICIs) have revolutionized treatment, yet primary and acquired resistance limit their efficacy (1–3). Established biomarkers such as PD-L1 expression, tumor mutational burden, and tumor-infiltrating lymphocyte status incompletely explain response heterogeneity (4–8), underscoring the need for novel mechanistic insights (5, 9, 10).

The gut microbiota has emerged as a key modulator of systemic immunity and ICI efficacy (8, 11, 12). Beyond the gut, the lung microbiota shapes cancer progression and immune responses through chronic inflammation and metabolic reprogramming (7, 13). More recently, the intratumoral microbiota has been recognized as an active modulator of the tumor immune microenvironment (14–16). These discoveries have propelled the concept of the “lung-gut axis”—a bidirectional network between gut and lung microbiomes mediated by metabolite exchange and immune cell trafficking (17, 18).

However, several critical questions remain unresolved. First, while gut microbiota-ICI associations are well documented, the extent to which these reflect causal relationships versus confounding remains contested. Second, the lung and intratumoral microbiota are often conflated despite occupying distinct anatomical niches with different functional implications. Third, the mechanistic pathways connecting these three microbial compartments remain incompletely defined. This review critically synthesizes current evidence on the tripartite regulatory network of gut, lung, and intratumoral microbiota in lung cancer immunotherapy. We evaluate evidence strength across study designs, identify key knowledge gaps, and propose a framework for future investigation.

## 2 The lung-gut axis and the tripartite microbiota

### 2.1 Lung microbiota: composition, distribution and ecological models

Contrary to the traditional view of a sterile lower respiratory tract, the human respiratory tract harbors a dynamic microbial ecosystem (19, 20). Microbial abundance and diversity decrease along the respiratory tract gradient: the upper tract is dominated by Firmicutes, Actinobacteria, and Proteobacteria, while the lower tract is enriched in Proteobacteria, Bacteroidetes, and Firmicutes (21, 22). Dickson and colleagues proposed three models of pulmonary microbiome ecology—the adapted island, environmental gradient, and complex adaptive system models—emphasizing the dynamic balance between microbial immigration and clearance (23).

In a healthy state, this balance ensures dynamic stability. Disease states can disrupt this equilibrium, leading to dysbiosis characterized by a reduction in beneficial commensals or the overgrowth of opportunistic pathogens (17, 22).

In lung cancer, this dysbiotic state is marked by immune cell infiltration, cytokine accumulation, and compositional shifts in the local microbiome (13). Dysbiotic communities may contribute to malignant transformation by secreting oncogenic metabolites (e.g., reactive oxygen species, secondary bile acids) that induce DNA damage or epigenetic alterations (24, 25). For instance, *Veillonella* metabolites have been shown to activate the PI3K/ERK pathway in lung epithelial cells, promoting malignant transformation (26). Clinically, the pulmonary microbiome profile of lung cancer patients diverges from those of healthy individuals, with elevated abundances of *Granulicatella*, *Thermus*, and *Streptococcus* emerging as potential diagnostic biomarkers (27).

Nevertheless, the roles of specific taxa in lung cancer remain debated. This likely reflects strain-level functional heterogeneity, spatial variations within the TME, and methodological discrepancies across studies. While compositional alterations are consistently observed, whether these shifts actively drive oncogenesis or primarily reflect the inflammatory milieu of the tumor-bearing lung requires further investigation.

### 2.2 Gut microbiota: composition and function

The gut microbiota, a complex community inhabiting the human intestine, includes bacteria, archaea, fungi, and viruses, with bacteria being the most dominant. This community, estimated at 4 × 10¹³ cells and encoding over 3 million genes—far exceeding the human genome—is often referred to as the “second genome” or “forgotten organ” (28, 29). In healthy adults, Firmicutes and Bacteroidetes constitute over 90% of the gut microbiota, with Actinobacteria and Proteobacteria representing minor populations (28, 30). The microbial composition also exhibits a gradient along the intestinal tract: the small intestine has a lower microbial density dominated by facultative anaerobes, while the colon hosts the highest density, primarily consisting of strict anaerobes (29).

The gut microbiota establishes a symbiotic relationship with the host, executing three core functions. First, metabolism and nutrition: It ferments dietary fibers, producing short-chain fatty acids (SCFAs) such as acetate, propionate, and butyrate. SCFAs not only fuel intestinal epithelial cells but also modulate systemic immune responses through multiple pathways (29, 31). Additionally, the gut microbiota participates in bile acid metabolism, vitamin synthesis, and tryptophan conversion (30), with over 10% of circulating metabolites in host blood originating from gut microbes (32). Second, intestinal barrier maintenance: Commensal bacteria protect intestinal barrier function by competitively excluding pathogens, secreting antimicrobial substances, promoting mucus production, and maintaining tight junction integrity (29, 33). Third, immune system development and regulation: The gut microbiota plays a crucial role in mucosal immune system maturation early in life. Its dysbiosis is associated with various diseases, including inflammatory bowel disease, obesity, type 2 diabetes, and cancer (30, 33).

### 2.3 The lung-gut axis: theoretical foundations

Historically, a functional relationship between the lung and intestine was noted in traditional medical systems (34). This conceptual parallel has found support in modern anatomical and immunological evidence, including shared embryonic origin from the endodermal foregut and common mucosal immunity (35, 36). Immune cell trafficking represents a key mechanism for this bidirectional lung-gut crosstalk: gut-derived lymphocytes, particularly α4β7 integrin and CCR9 expressing T cells, migrate to respiratory mucosal sites via the lymphatic and circulatory systems (18). Circulating microbial metabolites form another central pathway: gut microbiota-derived SCFAs, tryptophan metabolites (e.g., indole-3-propionic acid), and secondary bile acids enter systemic circulation via the portal vein and modulate alveolar macrophage polarization and T cell function in the lungs through G-protein-coupled receptors and aryl hydrocarbon receptors. Multi-omics studies from large human cohorts reveal that gut microbiota can explain nearly 30% of plasma metabolite variations (32). Neuroendocrine pathways also participate: the gut microbiota influences vagal nerve activity and enteroendocrine cell secretion of neuropeptides (e.g., glucagon-like peptide-1, peptide YY), regulating neurogenic inflammation and airway reactivity (37) (**Figure 1**).

**Figure 1:**
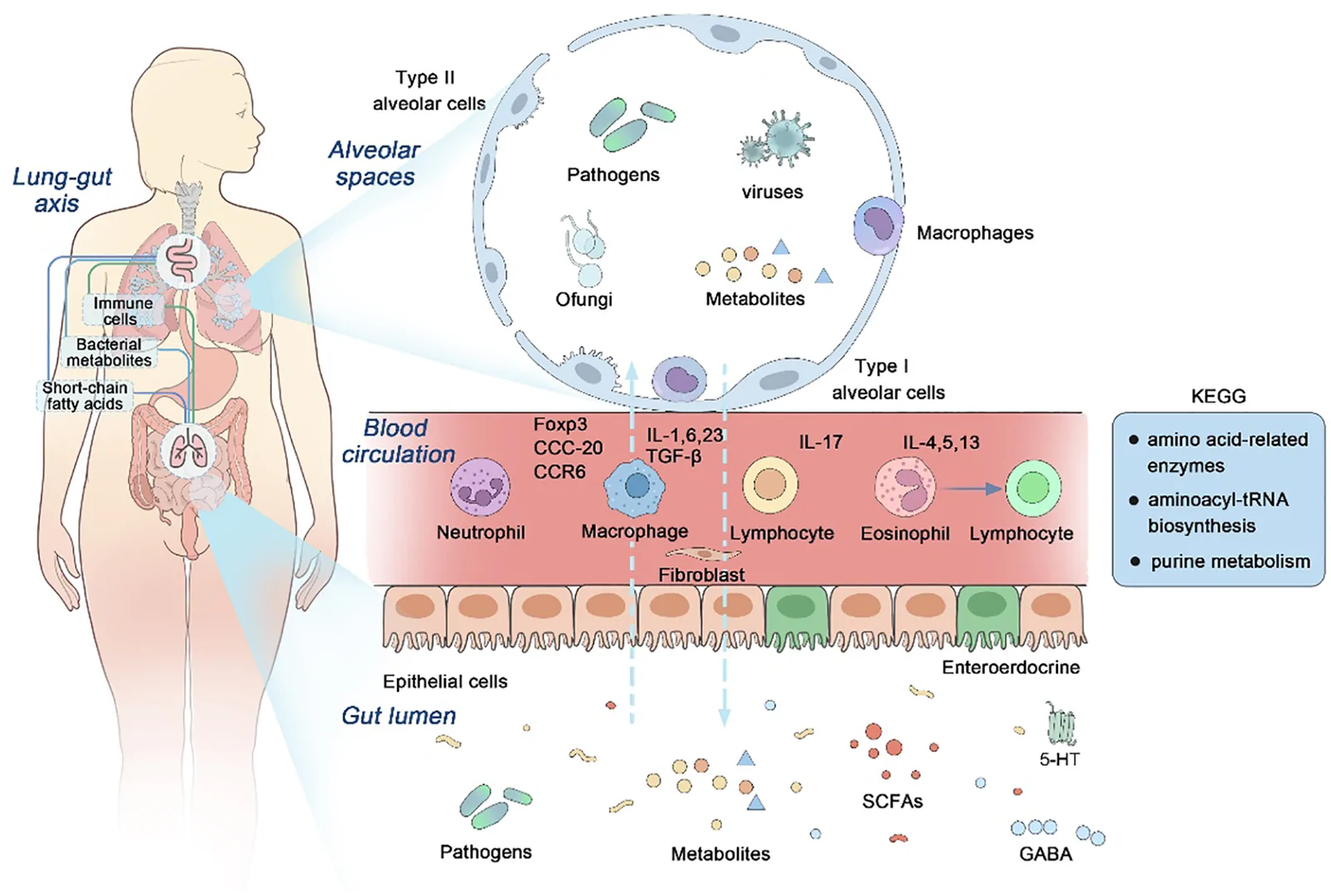
Schematic diagram of the bidirectional regulatory network of the lung-gut axis. The lung and intestine communicate through circulatory pathways, immune cell homing, and microbial metabolites. Gut-derived SCFAs, IPA, and secondary bile acids remotely regulate the pulmonary immune microenvironment, while lung-derived factors reciprocally influence gut microbiota composition.

### 2.4 Intratumoral microbiota: definition, characteristics, and evidence boundaries

Conventional wisdom once considered tumor tissues sterile. However, high-throughput sequencing has detected microbial DNA and viable bacteria within lung tumors, challenging the “sterile tumor” paradigm (38). The intratumoral microbiota refers to microbial communities residing within tumor tissues, potentially originating from local lung microbiota, gut translocation, or oral aspiration (39). Distinct from the lung microbiota inhabiting the bronchi and alveolar spaces, the intratumoral microbiota is intimately localized within the tumor microenvironment (TME), in direct contact with tumor and immune cells (40). **Table 1** summarizes the key distinguishing features between these two microbial compartments.

**Table 1:** Distinguishing features of lung microbiota and intratumoral microbiota in lung cancer.

Feature	Lung microbiota	Intratumoral microbiota
Anatomical localization	Bronchi, bronchioles, alveolar spaces	Within tumor tissues (tumor parenchyma)
Primary origin	Oropharyngeal aspiration, environmental exposure, microaspiration	Local translocation from adjacent lung tissue, hematogenous spread, oral aspiration, or gut translocation
Contact with tumor cells	Indirect (via secreted metabolites, immune cells, or inflammatory mediators)	Direct (physical contact with tumor cells and immune cells within the TME)
Primary functional role	Maintains immune homeostasis, defends against pathogens, regulates local inflammation	Directly modulates tumor cell behavior, immune evasion, and therapeutic response
Evidence maturity	Extensive preclinical and clinical studies; multiple mechanistic pathways established	Emerging field; largely correlational and descriptive; mechanistic evidence limited
Key methodological challenge	Contamination from upper respiratory tract during sampling	Low biomass and contamination from reagents, laboratory environment, and adjacent normal tissues (16, 38)

The lung cancer intratumoral microbiota is predominantly composed Proteobacteria, Firmicutes, and Actinobacteria, with enrichment of *Streptococcus* and *Veillonella* (41). Stage-specific heterogeneity has been observed, with *Bacteroides* enrichment in early-stage tumors correlating with favorable prognosis — an association requiring mechanistic validation (42). A 2026 study showed that intratumoral *Sphingobacterium multivorum* promotes Treg infiltration via CCL20 and CXCL8, suppressing CD8^+^ T cell function (43). More recently, two intratumoral microbial scores (KBUBe and OSAA) predicted survival and ICI response in NSCLC, outperforming PD-L1 expression (HR for OSAA: 0.491, p < 0.001) — a promising but preliminary step toward clinically actionable biomarkers (44). Multi-omics analysis has further revealed that intratumoral microbial structure closely associates with lactate metabolism patterns and immune status in lung squamous cell carcinoma (45).

Mechanistically, the intratumoral microbiota has been implicated in three modes of TME regulation (46): immune activation via cGAS-STING (47); immunosuppression through MDSC expansion (48), and PD-L1 modulation (40). These mechanisms are discussed in greater detail in Section 3.4, within the broader context of tripartite crosstalk. 

A methodological caveat is warranted when interpreting the above evidence. Tumor tissues are low-biomass environments, making contamination from reagents, laboratory environments, and adjacent normal tissues a persistent concern (16, 38). Many published studies lack adequate negative controls, and cross-cohort reproducibility remains limited. Thus, the evidence should be interpreted with appropriate caution: compositional findings and predictive scores are informative but require independent validation, while mechanistic insights remain largely preliminary. Whether observed associations reflect causal effects, contamination artifacts, or epiphenomena of tumor progression remains an open question. The intratumoral microbiota field is at an early stage, and prospective studies with rigorous controls are needed before it can be considered a therapeutically actionable target.

## 3 Orchestrating the lung cancer immune microenvironment

### 3.1 Lung microbiota: local immune regulation

The lung microbiota modulates local immune homeostasis through metabolites and pattern recognition receptors (PRRs) signaling. Alveolar macrophages and epithelial cells are the primary responders to microbial signals.

Upon microbial encounter, epithelial cells secrete antimicrobial peptides and mucus (49, 50). Alveolar macrophages, serving as innate immune sentinels, recognize microbe-associated molecular patterns (MAMPs) via Toll-like receptors (TLRs) and NOD-like receptors (NLRs), triggering NF-κB and MAPK pathways to initiate inflammatory responses (51, 52). Chronic low-grade microbial exposure can foster immune tolerance (53).

Microbial metabolites critically fine-tune this local immunological niche. Lower respiratory tract bacteria metabolize tryptophan into indole derivatives; depletion of indoleacetic acid exacerbates chronic inflammation and epithelial apoptosis (54, 55). Pulmonary SCFAs, derived from both local microbial activity and systemic circulation, maintain immune equilibrium by suppressing pro-inflammatory IFN-γ and IL-17A while promoting Treg differentiation (56).

Preclinical models underscore the therapeutic relevance of these mechanisms: aerosolized antibiotics can enhance NK and T cell anti-tumor activities by depleting immunosuppressive Tregs, whereas specific probiotics such as *Lacticaseibacillus rhamnosus* reverse local immunosuppression and curtail pulmonary metastasis (57).

Clinically, the pulmonary microbiome profile of lung cancer patients diverges from healthy cohorts. Elevated abundances of *Granulicatella*, *Thermus*, and *Streptococcus* have been reported as potential diagnostic biomarkers (27), though their specificity and sensitivity require further validation. Additionally, airway microbiota alterations have been associated with upregulation of the PI3K/ERK pathway — a finding that suggests a link between microbial composition and oncogenic signaling, but remains correlational (26). Whether these microbial shifts actively drive malignant transformation or merely reflect the inflammatory milieu of the tumor-bearing lung remains unresolved. The roles of specific taxa are debated, likely reflecting strain-level functional heterogeneity, spatial variations within the TME, and methodological differences across studies.

### 3.2 Gut microbiota: remote immune regulation via metabolites

The gut microbiota remotely shapes pulmonary immunity primarily through circulating metabolites. SCFAs (acetate, propionate, butyrate) modulate alveolar macrophage polarization and T cell function via G protein-coupled receptors and histone deacetylases (58). In preclinical models, SCFAs suppress tumor growth and enhance ICI efficacy, though butyrate exhibits a dualistic role—protective in most contexts but potentially promoting multidrug resistance (59). Notably, a 2025 study found that butyrate and propionate enhance PD-1 inhibitor therapy in NSCLC through the HDAC1/ID2/IL-12R pathway (60).

Tryptophan metabolites exert profound distal effects. In a pan-cancer study that included multiple tumor types, Jia et al. (61) demonstrated that *Lactobacillus johnsonii* and *Clostridium sporogenes* sporogenes synergistically produce indole-3-propionic acid, which maintains CD8^+^ T cell stemness via H3K27 acetylation at the *Tcf7* super-enhancer. While this mechanistic insight is relevant to lung cancer immunotherapy, lung-specific validation of this pathway remains an important area for future investigation. Secondary bile acids regulate Th17/Treg balance through farnesoid X receptor and TGR5 (62). TGR5 inhibition has been shown to enhance ICI efficacy in NSCLC by restraining M2 macrophage polarization and enhancing CD8^+^ cytotoxic T cells (63).

The above findings collectively paint a compelling mechanistic picture. However, before translating these insights into clinical practice, it is important to critically assess the strength and limitations of the underlying evidence. The metabolite-immune pathway evidence is mechanistically plausible but predominantly derived from preclinical model; human data remain largely correlative. A growing consensus in the field cautions that biological plausibility, however compelling, should not be equated with readiness for routine clinical implementation. Few studies have integrated microbiome, metabolomic, immune, and clinical outcome data within the same patient cohort — a critical gap that limits causal inference. Addressing this gap will require prospective, multi-omics studies designed to capture the full causal chain from microbial composition to metabolite production to immune modulation to clinical response.

### 3.3 The lung-gut axis: bidirectional regulatory mechanisms

The lung-gut axis operates as a highly coordinated, bidirectional interactive network mediated by immune cell trafficking, metabolite exchange, and neuroendocrine signaling. While the gut microbiota and its metabolites remotely govern pulmonary immune homeostasis, the lung microbiota and ensuing pulmonary immune responses reciprocally shape the intestinal landscape.

At the cellular level, this bidirectional dialogue leverages the common mucosal immune system. Gut-primed lymphocytes—particularly those expressing the α4β7 integrin and the chemokine receptor CCR9—can migrate through the lymphatic and circulatory systems to seed the respiratory mucosa, actively participating in local immune responses. Preclinical models confirm that gut dysbiosis triggers the aberrant migration of α4β7^+^ CD4^+^ T cells to the lungs, precipitating pulmonary inflammation (18). Conversely, pulmonary inflammation casts a shadow on intestinal immunity: respiratory syncytial virus (RSV) infection, for instance, induces lung-derived dendritic cells to migrate to gut-associated lymphoid tissue (GALT), modulating intestinal immune tone (32). This bidirectional homing, governed by precise integrin-adhesion molecule and chemokine-receptor axes, forms the core cellular bedrock of the lung-gut axis.

Metabolites act as critical informational vectors. In the anterograde pathway (gut-to-lung), gut-derived SCFAs, tryptophan metabolites (e.g., IPA), and secondary bile acids enter the systemic circulation via the portal vein, calibrating alveolar macrophage polarization and T cell activity via GPCR and aryl hydrocarbon receptor (AhR) signaling (17, 18, 61). In the retrograde pathway (lung-to-gut), metabolites originating from the pulmonary microbiota enter the systemic circulation to reciprocally alter gut microbial composition and metabolic output (64). A review highlighted that dynamic gut microbiota alterations in lung cancer patients are linked to disease progression and treatment, and longitudinal monitoring may aid early diagnosis and prognosis (65). The significant correlation between lung and gut microbiome profiles in lung cancer patients strongly suggests the existence of a self-perpetuating “gut-lung-gut” metabolic feedback loop.

Collectively, these bidirectional pathways—ranging from immune cell trafficking to metabolite anterograde and retrograde signaling—constitute the core mechanistic framework of the lung-gut axis. They illustrate that the gut and lung are not immunologically distinct compartments but rather a functionally integrated system connected by circulating metabolites and recirculating lymphocytes (7).

### 3.4 Tripartite crosstalk: connecting the three compartments

The gut, lung, and intratumoral microbiota do not operate in isolation. Here we synthesize the evidence for their inter-compartmental communication pathways and critically assess the strength of each connection (**Figure 2**).

**Figure 2:**
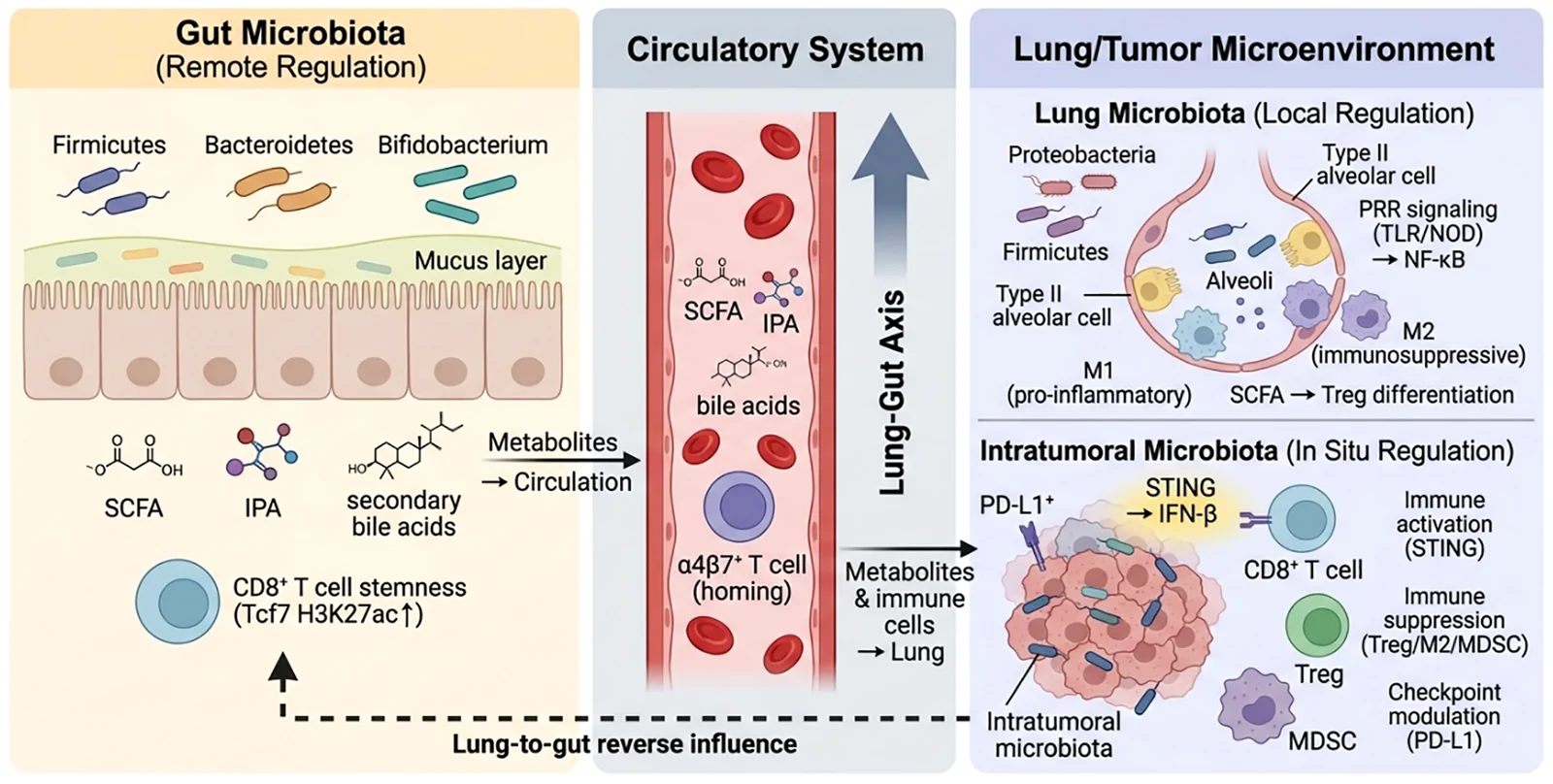
Tripartite mechanisms by which gut, lung, and intratumoral microbiota regulate lung cancer immunotherapy. The gut microbiota remotely modulates CD8^+^ T cell stemness via IPA-mediated epigenetic reprogramming. The lung microbiota locally regulates alveolar macrophage polarization through PRR signaling and SCFAs. The intratumoral microbiota exerts *in situ* effects via STING pathway activation, Treg/M2 polarization, and PD-L1 modulation.

Metabolite pathway (gut → circulation → lung/tumor). Gut-derived SCFAs, IPA, and secondary bile acids enter systemic circulation via the portal vein and reach pulmonary and tumor tissues, where they modulate CD8^+^ T cell function, macrophage polarization, and Treg differentiation (32, 61). This pathway is the best-supported mechanism, with metabolomic data showing that the gut microbiota accounts for nearly 30% of plasma metabolite variation (32). However, the direct demonstration of gut-derived metabolites reaching the tumor microenvironment in humans remains limited.

Immune cell homing pathway (gut ↔ lung). Gut-primed lymphocytes, particularly α4β7^+^ CCR9^+^ T cells, migrate through lymphatic and circulatory systems to respiratory mucosa (18). A 2025 study demonstrated that segmented filamentous bacteria antigen-mediated Th17 cells migrate from the gut to peripheral organs including the lung through a coordinated gut-lung immunological axis (66). This pathway is supported by animal models but human data are sparse.

Reverse pathway (lung/tumor → gut). Pulmonary inflammation can alter gut permeability and microbial composition. Ruan et al. (64) showed that lung-derived metabolites enter circulation to reciprocally alter gut microbial composition. This pathway is the least characterized, with most evidence derived from animal models of respiratory infection rather than lung cancer.

The concept of a tripartite network is intellectually appealing, but the empirical evidence supporting direct crosstalk among all three compartments remains incomplete. The gut-lung connection is relatively well-supported; the lung-intratumoral connection is confounded by anatomical proximity and sampling limitations; and the tumor-gut connection is speculative. A 2026 review in *Frontiers in Medicine* explicitly cautioned that “different microbial compartments are often discussed together despite carrying different evidentiary meanings” — gut data reflect systemic input, lung data reflect local conditions, and tumor data raise additional issues of low biomass and contamination (67). Our view is that the field should prioritize establishing direct causal links between specific microbial metabolites and defined immune outcomes in lung cancer patients before claiming a fully integrated tripartite network.

## 4 Modulation of lung cancer immunotherapy responses by the lung-gut axis microbiota

### 4.1 Microbial biomarkers for immunotherapy efficacy

The gut microbiome was established as a key determinant of ICI response in 2018. Gopalakrishnan, Routy, and Matson independently demonstrated that specific microbial features—including *Ruminococcaceae* enrichment, antibiotic sensitivity, and distinct taxa abundance—correlate with ICI outcomes across melanoma and NSCLC (68–70). These foundational findings prompted investigation into whether other microbial niches, particularly the lung microbiota, similarly shape anti-tumor immunity.

A prospective cohort study of 52 advanced NSCLC patients demonstrated that ICI responders have higher gut microbial α-diversity and enrichment of SCFA-producing bacteria (*Ruminococcaceae*, *Alistipes*, *Eubacterium)*, while *Rothia* and *Streptococcus salivarius* correlated with poor prognosis (71). Systematic reviews confirm *Faecalibacterium*, *Bifidobacterium*, and *Akkermansia* as indicators of favorable ICI responses (8).

Despite numerous positive associations, the clinical utility of microbiome biomarkers remains uncertain for several reasons. First, findings vary substantially across cohorts, likely due to geographic, dietary, and methodological differences (8). Second, most studies are retrospective and underpowered. Third, the predictive value of microbial signatures often does not exceed that of established clinical variables. A 2026 systematic review and meta-analysis found that while high α-diversity and *Akkermansia* enrichment were associated with improved outcomes, the effect sizes are modest and confidence intervals wide (72).

Recent high-resolution analyses suggest that strain-level specificity may improve predictive performance. Raziq et al. (73) identified strain-specific single-nucleotide polymorphisms that distinguished responders from non-responders, achieving predictive accuracies exceeding 90%. However, these findings require independent validation. Integrating microbial signatures from both feces and BALF may enhance predictive accuracy (15), but whether this approach is practically feasible in clinical settings remains to be determined(Y. 15).

### 4.2 Microbial mechanisms driving immunotherapy resistance

Antibiotic exposure is consistently associated with ICI resistance. Derosa et al. (74) first reported this association in NSCLC and renal cell carcinoma, and subsequent large-scale cohort studies have confirmed a 21% increased mortality risk with antibiotic exposure. A subsequent study of 199 NSCLC patients confirmed these findings (75). Large-scale cohort analyses show a 21% increased mortality risk with antibiotic exposure (76).

Mechanistically, antibiotic-induced dysbiosis may promote resistance through:

Metabolite depletion: The eradication of commensals drastically reduces the pool of circulating SCFAs and tryptophan metabolites (e.g., indole-3-propionic acid), directly undermining CD8^+^ T cell stemness and effector functions (4).

Immunosuppressive cell recruitment: Dysbiotic taxa induce IL-1β-dependent expansion and recruitment of myeloid-derived suppressor cells (MDSCs)(77).

Chemokine downregulation: Antibiotics suppress CXCL9 expression, crippling T cell infiltration and diminishing sensitivity to anti-PD-1 blockade (78).

A critical caveat must be emphasized: the majority of clinical data remain correlational. Whether dysbiosis drives resistance or is a consequence of advanced disease and treatment-related damage is unresolved. Prospective longitudinal sampling with mechanistic intervention studies are needed to establish causality. Current evidence supports avoiding non-essential antibiotics within 60 days of ICI initiation (79), but this recommendation is based on associative data, not definitive causal evidence.

### 4.3 Microbiome-targeted clinical interventions

Armed with a deeper mechanistic understanding, targeting the microbiome has emerged as a premier strategy to sensitize lung cancer to immunotherapy. Current interventional paradigms focus on four main pillars:

#### 4.3.1 Fecal microbiota transplantation

FMT represents a promising strategy to reverse ICI resistance (80). Proof-of-concept was established in melanoma by Baruch et al. (81) and Davar et al. (82). In NSCLC, the phase II FMT-LUMINate trial (n=20) reported an 80% ORR when combining FMT with anti-PD-1 therapy (83). A 2026 prospective study of 27 NSCLC patients with secondary PD-1 resistance found that FMT combined with PD-1 inhibitor rechallenge achieved an ORR of 29.6% and disease control rate of 63.0% (84). The TACITO trial in renal cell carcinoma provided additional evidence for FMT efficacy in a different tumor type (85).

The 80% ORR in the FMT-LUMINate trial is impressive but must be interpreted with extreme caution due to the small sample size (n=20) and lack of a control arm. The 29.6% ORR in the larger NSCLC study (n=27) is more modest and likely more generalizable. Strain-level genetic features, rather than taxonomic abundance, may dictate FMT success (86). However, optimal donor selection, dosing, and patient stratification remain undefined. Whether FMT efficacy in melanoma translates to NSCLC is uncertain given distinct tumor immunobiology. A 2026 systematic review and meta-analysis found that while FMT shows promise, evidence quality remains low and large randomized trials are urgently needed (87). Future clinical implementation requires standardized protocols and identification of patients most likely to benefit.

#### 4.3.2 Probiotics and synbiotics

A meta-analysis of 36 studies (n=2,746) reported a 45% ORR for probiotics combined with ICIs, with substantial heterogeneity across studies and cancer types; the NSCLC subgroup showed a 55% ORR (88). Komatsu et al. (89) linked ICI response to *Bifidobacteriaceae* enrichment, while Goto et al. (90) showed that *Clostridium butyricum* strain MIYAIRI 588 (CBM588) directly lyses lung cancer cells via Vγ9Vδ2 T cell expansion.

Most probiotic studies are retrospective with small sample sizes. The 55% ORR in NSCLC is promising but derived from heterogeneous, predominantly retrospective data. A 2026 meta-analysis of microbiome-modulating strategies found “exploratory subgroup signals” for probiotics in NSCLC but noted that evidence quality is low and prospective validation is needed (87). Non-targeted probiotic supplementation may interfere with ICI efficacy (88, 91). Until randomized controlled trials establish safety and efficacy, probiotic use in ICI-treated patients should be approached with caution and preferably within research protocols.

#### 4.3.3 Dietary interventions

A plant-based, fiber-rich diet fundamentally supports ICI efficacy (88). The intestinal fermentation of dietary fibers yields abundant SCFAs, which enhance anti-tumor immunity by inhibiting HDACs and activating GPCRs (59). Systematic reviews confirm that plant-based diets elevate gut α-diversity and selectively expand microbial consortia positively associated with ICI responsiveness (88).

#### 4.3.4 Precision antibiotic stewardship

The rationale for precision antibiotic stewardship in cancer patients receiving immunotherapy is supported by a growing body of evidence. Zitvogel et al.(Zitvogel et al., 2018) first proposed the concept that the gut microbiome acts as a “master regulator” of ICI efficacy, arguing that antibiotic-induced dysbiosis directly impairs anti-tumor immune responses. Since then, multiple clinical studies have substantiated this observation. A meta-analysis confirms that antibiotic use within one month prior to ICI therapy increases mortality risk by 21% (88, 92, 93). This is driven by dendritic cell impairment and CXCL9 downregulation in the TME. Consequently, avoiding non-essential antibiotics within 60 days prior to ICI therapy is a critical clinical guideline. For patients requiring unavoidable antibiotic therapy, concurrent rescue strategies—such as targeted probiotics or post-treatment FMT—should be strongly considered to mitigate microbial damage (88, 91, 94). **Figure 3** summarizes the integrated workflow from multi-omics profiling to microbiota-targeted interventions (**Figure 3**).

**Figure 3:**
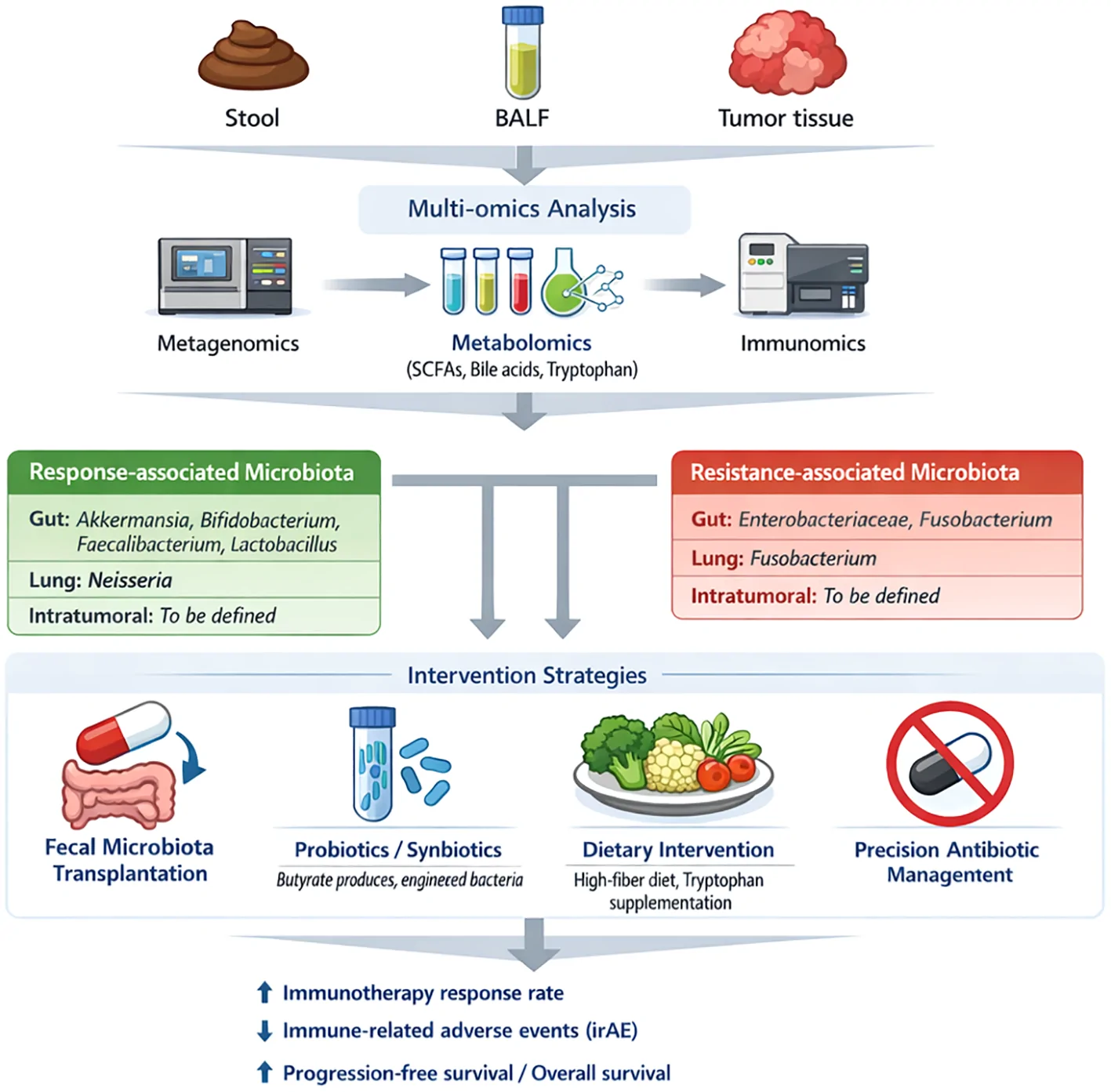
Integrated illustration of microbiota-based biomarkers and intervention strategies for lung cancer immunotherapy. Multi-omics analysis of stool, BALF, and tumor tissue identifies response-associated (green) and resistance-associated (red) microbial signatures. Targeting these features through FMT, strain-specific probiotics, dietary fiber, and antibiotic stewardship aims to enhance immunotherapy response rates and reduce immune-related adverse events.

### 4.4 The multi-omics paradigm

Integrating multi-omics data provides a revolutionary framework for decoding the complex regulatory networks of the lung-gut axis during immunotherapy. The recently proposed “microbiome-immune-tumor axis” concept emphasizes that fusing metagenomic, metabolomic, and immunomic data can systematically unravel the multidimensional crosstalk between microbial metabolites, host immune responses, and the TME (95). This integrative paradigm lays the theoretical groundwork for constructing personalized microbiome-immune predictive models and identifying precise interventional targets. Future translational efforts must leverage multi-omics coupled with machine learning algorithms to actualize precision microbiome-targeted therapies. 

## 5 Challenges and future perspectives

While emerging research underscores the potential of the lung-gut axis in modulating lung cancer immunotherapy, several critical bottlenecks impede immediate clinical translation.

First, the majority of current clinical cohorts rely on correlative analyses, precluding the definitive establishment of causal relationships between microbial shifts and therapeutic enhancement. Bridging this gap necessitates rigorous functional validation utilizing germ-free animal models, engineered microbial consortia, and well-designed FMT intervention studies.

Second, the profound spatial heterogeneity between the intestinal and pulmonary microbiomes means that single-site sampling fails to capture the intricate reality of microbial crosstalk. The adoption of high-resolution spatial transcriptomics and single-cell sequencing is imperative to map the precise *in situ* localization and functional state of microbes within the TME.

Third, isolated single-omics approaches are fundamentally inadequate for deciphering the complex microbiome-metabolism-immune-tumor network. The pivotal breakthrough will lie in the seamless integration of multi-omics datasets powered by advanced artificial intelligence and machine learning architectures.

Moving forward, the field should actively pivot towards experimental causal validation, multidimensional spatial mapping, and AI-driven multi-omics integration. By establishing robust causal frameworks, developing personalized predictive algorithms, and executing large-scale, prospective randomized controlled trials, strategies such as FMT, precision probiotics, and dietary interventions can transition from the bench to bedside. Ultimately, deciphering and manipulating the lung-gut axis holds the key to establishing a novel, transformative paradigm for sensitizing lung cancer to immunotherapy.
